# Trends in women’s height and the effect of early childbearing on height retardation: An analysis of the height of Bangladeshi women born between 1974 and 1998

**DOI:** 10.7189/jogh.13.07006

**Published:** 2023-09-29

**Authors:** M Moinuddin Haider, Nahid Kamal, Shusmita Khan, Md Mahabubur Rahman, Md Nayem Dewan, Sadman Sowmik Sarkar, Sabit Saad Shafiq, Nurul Alam

**Affiliations:** 1 International Centre for Diarrhoeal Disease Research, Dhaka, Bangladesh; 2 PopDev Consultancy Ltd., London, UK; 3 Data for Impact, University of North Carolina at Chapel Hill, USA; 4 Bangladesh Bank, Dhaka, Bangladesh

## Abstract

**Background:**

Depending on race, ethnicity, and region, genetic variants determine human height by 65% to 80%, while the remaining variance of 20% to 35% is influenced by nutrition and other individual or environmental exposures in the early years of life. An improvement in nutrition and health in the early years in a population underprivileged in health and nutrition will likely increase the group's average height. Due to outstanding improvements in these areas in recent decades, we hypothesised that the average height of Bangladeshi women has increased. Moreover, because pregnancy at an early age affects women’s health and nutrition, we hypothesised that women who began childbearing early would experience growth retardation compared to women who had pregnancies at a later age.

**Methods:**

We used data from five national surveys conducted between 2004 and 2018 that collected height data from ever-married women aged 15-49 years. We analysed the height of women aged 20-29 years (born between 1974 and 1998) and examined the mean height by birth years, age at first birth (AFB), economic status, religion and region. We conducted multiple linear regression models, controlling for the differential effects of the socio-demographic characteristics on women’s height over time and by AFB.

**Results:**

The average height of women born between 1974 and 1998 significantly increased by 0.03 cm annually, with fluctuations between 150.3 and 151.6 cm. We also found an association between age at childbearing and height in adulthood – women who began childbearing before age 17 were approximately one centimetre shorter in adulthood than those who began childbearing at a later age.

**Conclusions:**

We found evidence of an increasing trend in women’s height in Bangladesh and an inhibiting effect of early teenage childbearing on attaining the potential growth of women. The findings call for further studies to investigate early childbearing and its consequences on women's and their children's growth in diverse settings, considering socio-cultural customs influencing early marriage and childbearing.

Height is associated with health and well-being, like nutritional status, cognitive performance, life expectancy, average income, etc., with intergenerational linkage [1-7]. This biological trait varies across human populations; for example, the Dutch are arguably the tallest, Sudanese men are as tall as Dutch women, while Sudanese women are taller than East Timorese men [8-10]. Human height not only exhibits cross-sectional differences by race, ethnicity, and sex, but has also evolved over time. The skeletal length of prehistoric humans in Europe shows fluctuations over the decades [11]. Change in human height is also evident in recent history – a few Asian national populations (e.g. South Koreans, Iranians, and Japanese) have become taller in the past century; in contrast, seven sub-Saharan countries recorded a decline in population height [12-14].

Adult human height is a complex function of hundreds of genetic variants, along with childhood and adolescent nutrition and morbidities; of these factors, genetic variants determine the height by 65%-80% depending on race, ethnicity, and region [15-17]. For example, heritability determines the height of the Australian and Finnish populations by 75%-80%, but that of the Chinese population by only 65% [16-18]. Nutrition is perhaps the strongest factor after heritability [19]. Malnutrition and morbidities in childhood and adolescence are likely to disrupt growth in height in full biological potential.

The common adverse exposures that affect childhood nutrition and morbidities include poverty, food insecurity, morbidities, lack of access to health care, polluted and unhygienic environments, etc. [19]. Pregnancy during adolescence – when a girl has not fully developed physically – can compromise her health and prevent her from reaching her full height in adulthood [12]. Her nutritional status and health may be further compromised during lactation and childcare. Alongside nutrition, household socioeconomic status, one of the most powerful predictors of health and well-being, is also closely associated with height [20]. The examples of South Koreans and Japanese may better explain the context – the increase in the height of the two populations in the past four to five decades is largely attributable to dietary and nutritional improvement [13]. This discussion provides a conceptual understanding that a population’s nutrition improvement, which is a function of socioeconomic, dietary, and other improvements, may increase the population's average height.

Bangladesh is a densely populated country with a long history of poverty and food insecurity, lack of access to improved sanitation and safe drinking water, high child mortality and malnutrition, and marriage and pregnancy in adolescence [21-30]. Nevertheless, since its independence in 1971, it has emerged as an exemplary nation from the global south, having successfully tackled many of these adversities [31], and has seen remarkable increases in gross domestic product per capita, per capita calorie intake, protein consumption, access to improved water and sanitation facilities, as well as declines in maternal and child mortality, morbidity, and undernutrition, early marriage, and childbearing [27,30,32-40].

Based on factors affecting human height, the possibility of a population height increase under certain circumstances, and Bangladesh’s recent development in economy, food security, health, and nutrition in recent decades, we would expect that the average height of the population, especially women, has been increasing. For example, an ongoing follow-up study in Matlab, Bangladesh, found 71% of daughters taller than their mothers [41]. However, the finding is based on a small homogeneous population from the South-Eastern region of Bangladesh, and there are no other studies that examined changes in human height in Bangladesh.

## Study objectives

This study had two objectives. For the first one, we hypothesised that the average height of Bangladeshi women has increased, so we aimed to examine trends in Bangladeshi women’s height over time. Regarding the second objective, we know that a girl may grow in height until approximately the age of 16 years [42]. Pregnancy during this time may affect her growth adversely because she shares nutrients with the foetus [43], while pregnancies during the age of 10-19 years (rather than ages 20-24) go through more complications and other health hazards [15]. Therefore, we hypothesised that women who began childbearing early, especially by the age 16 or 17 years, were shorter than others. We studied the case of Bangladesh, as early marriage and childbearing are common in the country – around 16% of girls are married by the age 15 and 13% give birth by the age 16 [44,45].

## METHODS

**Figure 1:**
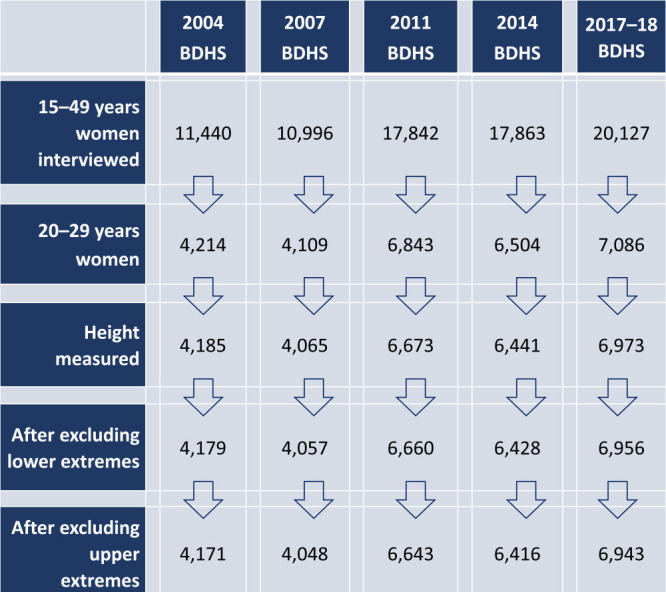
Data flow diagram for women’s height, 2004, 2007, 2011, 2014, and 2017-2018 BDHS.

We conducted an observational study based on data from five rounds of the nationally representative Bangladesh Demographic and Health Surveys (BDHS) conducted in 2004, 2007, 2011, 2014, and 2017-2018. The surveys collected birth history, anthropometric data, and other demographic and health data from all ever-married women ages 15-49-year living in the sampled households. All five surveys adopted a two-stage cluster sampling design (see **Figure 1** for flow diagram of the data used in this study).

Our outcome measure was women’s height. The five BDHSs included in this study used the Shorr Board to measure the height of every woman thrice, taking the average of three measures as the height of the individual.

We included women aged 20-29 years on the day of survey, because females, according to their anatomy, grow in height until 16 and begin shrinking after 30 years [42,46]. Thus, we excluded women aged under 20 and those aged 30 years and above from the analysis. The 20-29 years age range ensured that women had reached their maximum height and had not begun shrinking. We excluded 416 of the 28 754 women aged 20-29 years due to missing height data, an additional 57 as the height data were below the lower extreme (-3<standard z-score) and 59 as height data were above the upper extreme (standard z-score>+3). Women’s inclusion criteria do not meet Deaton’s discussion on the South Asian women’s age at reaching adult height (until early 20s), because he neither determined any age cut point nor cited other studies from where we can get detailed information [42,47]. We used height as a continuous variable in the centimetres scale.

We examined birth year (BY) and women’s age at first birth (AFB) as two primary variables. For BY, women aged 20-29 surveyed in the five BDHSs were born between 1974 and 1998. We used this variable (BY) as both continuous and categorical, as applicable at different examinations. BY is the proxy for economic, health, nutrition, and other developments over time, during which the different women cohorts in this study experienced differently. Regarding AFB, we categorised it into eight groups: <15 years, 15, 16, 17, 18, 19, 20+, and “no child” (no child: women who never gave birth). The categorisation was useful for understanding any nonlinear relation of the outcome with the covariates.

Other covariates were household wealth quintiles (HWQs) (poor – bottom two HWQ, middle – third HWQ, and rich – upper two HWQs, region (West – Khulna, Rajshahi, and Rangpur divisions, Central – Dhaka, Mymensingh, and Barishal divisions, and East – Chattogram and Sylhet divisions), and religion (Muslim and non-Muslim (mostly Hindu)).

We used cross-sectional survey data and examined the height of women who had already grown to their maximum height. We knew the household wealth index and region of the women’s husband’s or in-laws’ households, as they were interviewed there. However, how tall a woman grew depended on her childhood and adolescence nutrition and other factors, which in turn largely depend on the characteristics of the parental household where she grew up. We used the HWQs of the woman’s husband’s or in-laws’ household as a proxy of her parent’s household because the bride and groom tend to come from a similar economic class in Bangladeshi culture. Unpublished data from the Matlab Health and Demographic Surveillance System (HDSS) showed that eight in ten of the brides and grooms married in 2014 in the HDSS area came from the same HWQs (Appendix A1 in the **Online Supplementary Document**). The bride and groom have an identical religious affiliation in Bangladesh, with rare exceptions. We also assumed that most women’s parental and in-laws’ households were in the same geographical region.

### Statistical analysis

We described women’s height using minimum and maximum values, medians, means, and standard deviations, and examined the normality of height data using graphical methods (density plot, Q-Q plot, probability plot, and symmetry plot). We also analysed height by the socio-demographic characteristics in numbers and percentages. We determined the required sample size to estimate the mean height for each BY.

For our first objective, we estimated the mean height of women by their BYs (grouped) and fitted a simple linear regression (SLR) model using equation 1 to understand the changing pattern in the average height by BYs. Based on our theoretical understanding, AFB, HWQs, region of living, and religion are likely to affect women’s height [48-50]. Therefore, we estimated the average height of women by these characteristics and also controlled the characteristics for women’s height in a multiple linear regression (MLR) model, as specified in equation 2.

We examined women’s average height by AFB under objective two. The SLR specified in equation 3 examined whether women’s average height was different by the AFB. The effect of other characteristics – BY, HWQ, region, and religion – were controlled in the MLR specified in equation 2.

1) *Y_i_* = a + b*BY_i_* + ε*_i_*

2) *Y_i_* = a + b*BY_i_* + c*AFB_i_* + Σg*_k_X_ki_* + ε*_i_*

3) *Y_i_ =* a + c*AFB_i_* + ε*_i_*

4) *Y_i_* = a + d*BY*2*_i_* + e*AFB*2*_i_* + f(*BY*2*_i_ x AFB*2*_i_*) + Σg*_k_*X*_ki_* + ε*_i_*

Here, *Y_i_* represents the women’s height (discrete continuous), *BY_i_* the birth year of individual *i* (discrete continuous), *AFB_i_* the age of firth birth of individual *i* (categorical), *BY2_i_* the birth year of individual *i* (categorical), *AFB2_i_* the age at the first year of individual *i* (categorical, the categories are different than the categories of *AFB_i_*). *BY2_i_ × AFB2_i_* represents the interaction of birth year and age at first birth, with *BY2_i_* representing the birth year of individual *i* (categorical). *X_ki_* represents the vector of background covariates likely to influence height and ε*_i_* the residual for individual *i*. The corresponding coefficient a is the intercept, b for *BY_i_*; c for *ABF_i_,* d for *BY2_i_ × AFB_i_*, and e for *ABF2_i_*

We also analysed the difference in height by AFB for women born at different times, using the MLR specified in equation 4, where BY and AFB appeared in interaction. Besides reporting the interaction coefficients between BY and AFB, we estimated the predicted average of each interaction category.

The complex design of the five surveys made weighted analysis essential. We incorporated the survey design by using the Demographic and Health Survey (DHS) program-provided survey weights in the analysis. The BDHS had separate survey weights for each specific survey. Analysis of two or more survey data together (pooled) required adjustment in the survey weight to take care of normalisation done in each survey for the total population in the country in that survey year. The descriptive and regression analyses performed used the adjusted weights for pooled analysis (**Table 1**). We used Stata, version 15 (StataCorp LLC, College Station, Texas, USA) for data analysis.

**Table 1:** Adjustment of survey weights for pooled data analysis of the 2004, 2007, 2011, 2014, and 2017–2018 BDHS

Index (i)	BDHS year	Total popu-lation in the country (million)*	Propor-tion of ever married women ages 15-49 y in the popu-lation†	Total ever- married women in the country (million)	Ever- married women inter-viewed	Proportion of women inter-viewed	Women to be inter-viewed in survey i to have same proportion of women inter-viewed in all surveys (base year 2017–2018)	Factors to be multi-plied by weights for ever-married women respondents
(1)	(2)	(3)	(4)	(5) = (3) × (4)	(6)	(7) = (6)/((5) × 1000000)	(8) = ((7)1/(7)i) × (6)i	(9) = (8)/(6)
1	2017–2018	161	0.24	38.64	20127	0.000521	20127	1.000000
2	2014	155	0.24	37.20	17863	0.000480	19377	1.084752
3	2011	149	0.23	34.27	17749	0.000518	17851	1.005732
4	2007	143	0.22	31.46	10996	0.000350	16387	1.490273
5	2004	137	0.23	31.51	11440	0.000363	16413	1.434711

*Source: United Nations World Population Prospects [51]. Population average of two years is used where the survey spanned over two calendar years.

†Source: Bangladesh Demographic Health Survey [52]. Estimated as (number of interviewed ever-married women ages 15-49 y)/(number of recorded household members).

## RESULTS

### Sample characteristics

We included 28 221 women (weighted 32 820) in the analysis, with the total number of participants from each survey ranging between 4048 and 6943 (**Figure 1**). Except for 1974-1976 and 1998, the numbers of women by BYs were large enough to estimate average height by BYs (**Table 2**). The participants’ height ranged from a minimum of 133.9 cm to a maximum of 168.1 cm, with a mean of 150.9 cm and a median of 150.8 cm (Table S2 in the **Online Supplementary Document**). The density plot, Q-Q plot, probability plot, and symmetry plot of height indicated that it was normally distributed. One in three (35%) of the women had their first birth by age 16, 37% between ages 17 and 19, 20% at or after age 20, and 9% had no child. Near two-fifths of the women came from the bottom two HWQs, one-fifth from the middle HWQ, and the other two-fifths came from the upper two HWQs (**Table 3**).

**Table 2:** Frequency distribution of the women, by single age and birth years

	Age at survey											
**Birth year**	**20**	**21**	**22**	**23**	**24**	**25**	**26**	**27**	**28**	**29**	**Total**	Required n = (zσ/0.5)^2^*
1974										272	272	407
1975									295	99	394	407
1976								301	92		393	444
1977							323	81		268	672	456
1978						320	98		273	78	769	431
1979					332	111		279	98		820	438
1980				298	99		287	98			782	413
1981			365	91		306	110			115	987	478
1982		304	111		298	107			126	476	1422	450
1983	353	120		297	112			135	504		1521	427
1984	106		310	126			154	518		189	1403	419
1985		274	144			144	545		204	463	1774	430
1986	256	158			139	568		205	451		1777	435
1987	169			126	534		199	429		11	1468	435
1988			178	578		204	493		17	638	2108	434
1989		117	554		191	464		20	614	58	2018	429
1990	124	458		201	444		25	619	49		1920	460
1991	550		170	442		21	618	52			1853	414
1992		167	483		21	630	77				1378	411
1993	187	405		13	621	58					1284	426
1994	425		21	588	81						1115	395
1995		22	635	63							720	455
1996	23	622	66								711	427
1997	535	60									595	454
1998	65										65	456

*z = 1.96 is the two-tailed z-value for 95% level of significance, σ the standard deviation of the outcome variable obtained from prior study, and d the assumed deviation between the mean outcome (here height) of sample and population. We used σ for estimating n of year t is the σ calculated from height data of year t-1, except for 1974. σ for 1974 is calculated from 1974 data.

**Table 3:** Percentage distribution of women, by socio-demographic characteristics and surveys

Background characteristics	Number of women (unweighted)	Weighted number	Weighted percentage
**Age at first childbirth (in years)**			
<15	2776	3458	10.5
15	2868	3583	10.9
16	3569	4264	13.0
17	3562	4149	12.6
18	3618	4209	12.8
19	3185	3631	11.1
20+	5886	6495	19.8
No child	2757	3030	9.2
**Household wealth quintiles**			
Bottom 2	10 302	12629	38.5
Middle	5336	6357	19.4
Upper 2	12 583	13834	42.2
**Religion**			
Other	2753	3062	9.3
Islam	25 468	29757	90.7
**Region**			
West	10 743	11575	35.3
Central	9213	12834	39.1
East	8265	8411	25.6
**Survey year**			
2004	4171	5989	18.2
2007	4048	6034	18.4
2011	6643	6733	20.5
2014	6416	7084	21.6
2017–18	6943	6980	21.3
**Total**	28 221	32820	100.0

**Figure 2:**
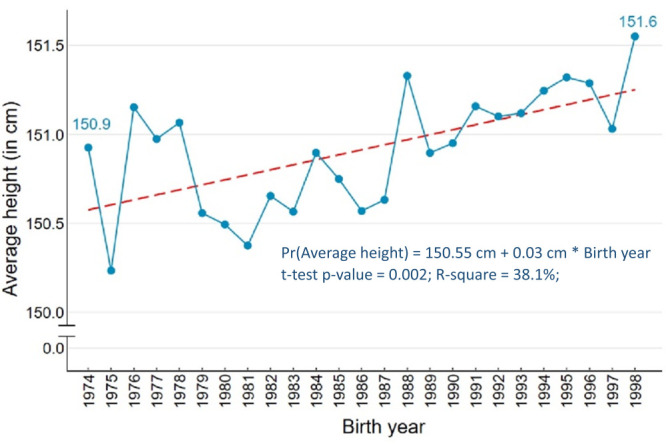
Average height of women born between 1974 and 1998, by birth year.

The average height of women born between 1974 and 1985 was 150.6 cm, increasing to 151.6 cm among women born between 1986 and 1998 (data not shown). From the year-by-year average, we also saw a gradual increase in women’s height through fluctuations, which seemed natural. The SLR-based fitted line also showed a 0.03 cm average annual increase, meaning that women born in a year were 0.03 cm taller than those born in the previous year (statistically significant) (**Figure 2**).

**Figure 3:**
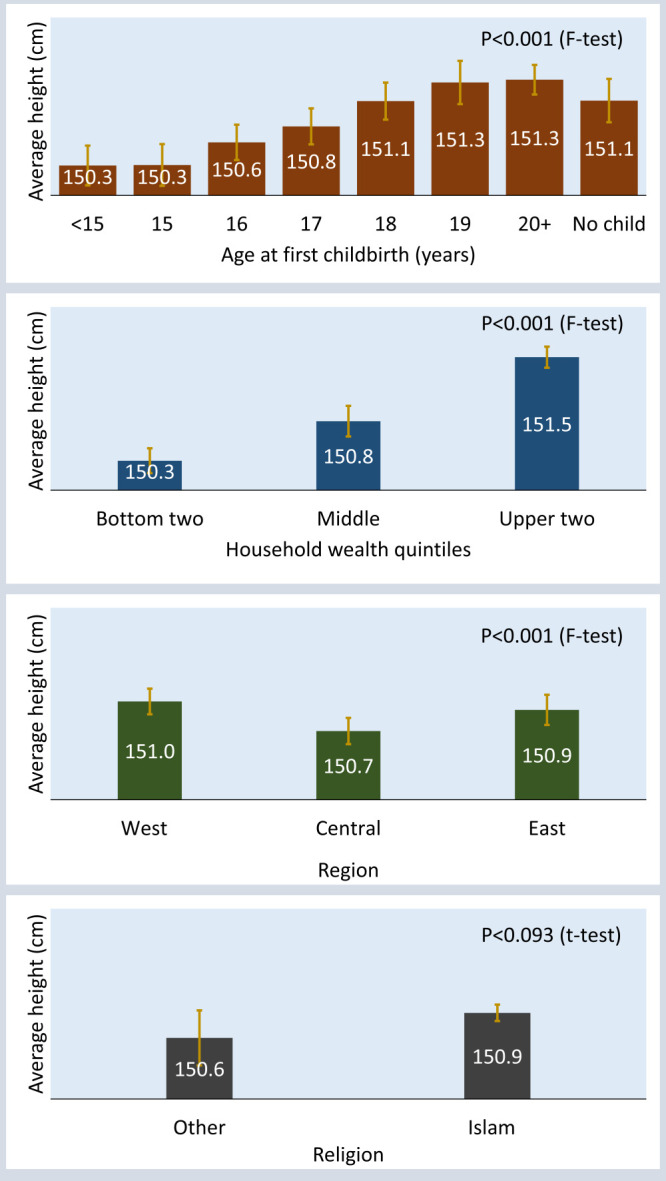
Average height of women born between 1974 and 1998, by socio-demographic characteristics.

We found that women in different socio-demographic groups were different in height (**Figure 3**). Women who gave birth at or before age 15 were 150.4 cm tall (summary not shown). Those who had delayed the first birth until age 17 were taller (150.8 cm). Women who gave birth at age 18 or later had a similar height of 151.2 cm (summary not shown). Women from the highest two HWQs were 1.2 cm taller than those from the lowest two HWQs (151.5 cm vs 150.3 cm). The average height of the women in the western, central, and eastern regions was 151.0 cm, 150.7 cm, and 150.9 cm, respectively. The difference in women’s height between Muslims (150.9 cm) and non-Muslims (150.6 cm) was 0.3 cm. AFB, HWQs, and region were significantly associated with women’s average height; however, religion was not.

The bivariate analysis found socio-demographic differentials in women’s height. Therefore, we controlled these characteristics in MLR models to examine the change in height over time (model 3 in **Table 4**). We found a 0.03 cm annual increase in women’s height over the study period, 1974 to 1998 (*P* < 0.001).

**Table 4:** Association of height with birth year, age at first birth, and socio-demographic variables: Results from multiple linear regressions

	Model 1			Model 2			Model 3		
**Covariates**	**Coeff.**	***P*-value (*t* test)**	**95% CI**	**Coeff.**	***P*-value (*t* test)**	**95% CI**	**Coeff.**	***P*-value (*t* test)**	**95% CI**
**Birth year**	0.03	<0.001	0.02,0.05	-	-	-	0.03	<0.001	0.01, 0.04
**Age at first birth**									
<15 years	-	-	-	Ref.	-	-	Ref.	-	-
15	-	-	-	0.05	0.766	0.28, 0.38	0.03	0.856	-0.30, 0.37
16	-	-	-	0.26	0.078	0.03, 0.54	0.20	0.173	-0.09, 0.49
17	-	-	-	0.42	0.005	0.12, 0.71	0.32	0.039	0.02, 0.62
18	-	-	-	0.70	<0.001	0.41, 0.99	0.55	<0.001	0.26, 0.85
19	-	-	-	0.87	<0.001	0.53, 1.21	0.67	<0.001	0.32, 1.01
20+	-	-	-	0.96	<0.001	0.69, 1.24	0.72	<0.001	0.44, 1.00
No child	-	-	-	0.70	<0.001	0.37, 1.03	0.35	0.045	0.01, 0.69
**Household wealth quintiles**									
Bottom 2	-	-	-	-	-	-	Ref.	-	
Middle	-	-	-	-	-	-	0.37	<0.001	0.17, 0.58
Upper 2	-	-	-	-	-	-	1.05	<0.001	0.88, 1.23
**Religion**									
Other	-	-	-	-	-	-	Ref.	-	-
Islam	-	-	-	-	-	-	0.33	0.028	0.04, 0.63
**Region**									
West	-	-	-	-	-	-	Ref.	-	-
Central	-	-	-	-	-	-	-0.48	<0.001	-0.67, -0.29
East	-	-	-	-	-	-	-0.30	0.004	-0.51, -0.10
**Constant**	150.46	<0.001	150.25, 150.68	150.34	<0.001	150.12, 150.56	149.58	<0.001	149.15, 150.02
Observations used	28 221	-	-	28 221	-	-	28 221	-	-

Coeff. – Coefficient, CI – confidence interval

The MLR model also provided statistical evidence of the relationship between height and each of the socio-demographic control variables. Women who gave birth at age 15 or 16 were of similar height as those who gave birth before age 15. However, giving birth at age 17 resulted in being 0.32 cm taller than giving birth before age 15. It was 0.55 cm for women giving birth at age 18 and above 0.67 cm for women giving birth at age 19 or later. Women with no child were 0.35 cm taller than women giving birth before age 15 (model 3 in **Table 4**).

The MLR model, after controlling for background characteristics, showed a significant difference between the height of non-Muslim and Muslim women, which was not the case in the bivariate analysis. The AFB, HWQs, and regional differentials in women’s height remained statistically significant, as they were in the bivariate analysis (model 3 in **Table 4**).

**Figure 4:**
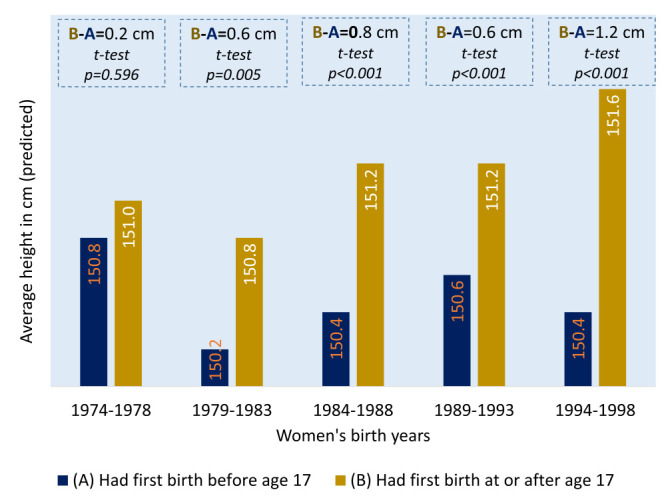
Women’s average height by age at first birth and women’s birth cohort.

We found giving birth before age 17 was negatively associated with women’s height (**Figure 3** and **Table 4**), while the interactions between BY and AFB were also significant (**Table 5**). Born in 1974-1978, women giving birth before age 17 were similar (difference = 0.2 cm) in height compared with women giving birth at or after age 17. However, among women born in the following five years, giving birth before age 17 made women shorter by 0.6 cm compared with those who gave birth at or after age 17. In the following three five-year cohorts, these differences were 0.8 cm, 0.6 cm, and 1.2 cm, respectively. The differences were statistically significant for all birth cohort women, except those born in 1974-1978 (**Figure 4**).

**Table 5:** Association of height with birth year, age at first birth, and socio-demographic variables: Results from multiple linear regressions

Correlates	Coefficient	*P*-value (*t* test)	95% CI
**Birth year**			
1974-1978	Ref.	-	-
1979-1983	-0.58	0.018	-1.06, -0.10
1984-1988	-0.48	0.038	-0.93, -0.03
1989-1993	-0.25	0.295	-0.70, 0.21
1994-1998	-0.40	0.177	-0.98, 0.18
**Age at first birth**			
Under 17	Ref.	-	-
17 and above	0.01	0.980	-0.47, 0.48
No child	0.03	0.964	-1.38, 1.44
**Birth year/age at first childbirth (interaction)**			
1979-1983, 17 and above	0.33	0.258	-0.24, 0.91
1979-1983, No child	<-0.01	0.996	-1.57, 1.57
1984-1988, 17 and above	0.65	0.022	0.10, 1.20
1984-1988, No child	0.13	0.863	-1.38, 1.65
1989-1993, 17 and above	0.47	0.090	-0.07, 1.02
1989-1993, No child	0.46	0.541	-1.02, 1.95
1994-1998, 17 and above	1.03	0.003	0.34, 1.72
1994-1998, No child	0.58	0.470	-0.99, 2.14
**Household wealth quintiles**			
Bottom two	Ref.	-	-
Middle	0.39	<0.001	0.18, 0.60
Upper two	1.09	<0.001	0.92, 1.27
**Religion**			
Other	Ref.	-	-
Islam	0.31	0.042	0.01, 0.61
**Region**			
West	Ref.	-	-
Middle	-0.48	<0.001	-0.67, -0.29
East	-0.29	0.005	-0.50, -0.09
**Constant**	150.38	<0.001	149.88, 150.89
Observations	28 221	-	-

CI – confidence interval

## DISCUSSION

We aimed to answer two main research questions: whether the height of Bangladeshi women had increased over time and whether there was any association between early childbearing and height in adulthood. We found that, although the average height of Bangladeshi women has increased over time, some sub-group differences persisted, with Muslims, those from higher HWQs, and those residing in western divisions being relatively taller. The most significant difference was found to be AFB. Women giving birth while still growing (≤16 years) were more likely to have retarded growth later in life compared with women giving birth afterward.

The consistent increase in the average height of women born between 1980 and 1998 was likely influenced by Bangladesh’s socioeconomic development. Progress in nutrition, medicine, health care, water, sanitation, and hygiene, along with steady improvement in female education possibly contributed to the increasing trend in height. However, nutrition and income are not always related to height, as seen in certain African countries where women are the tallest in the world. Deaton [47] demonstrated that, between 1950 and 1980, women’s height was positively associated with the national income, with the average female height in Bangladesh, India, and Nepal being the lowest in the world and where the per capita gross domestic product was also the lowest.

Women in South Asia have historically been short, where child stunting – although lower than before – remains among the highest in the world, so that the next generation of women is more likely to continue to be short. It has been hypothesised that the reason for women’s short height in South Asia has been their carbohydrate-dense diet, since a more balanced diet with a higher percentage of fats and animal-based foods is generally associated with being tall. Cultural practices stemming from gender bias in favour of male children further compromise women's health in South Asia.

Although an increase in mean height may indicate progress in improving living standards, such improvements have not occurred equitably in many countries [12]. In our study, religious sub-groups, namely non-Muslims (mostly Hindus) seemed disadvantaged. Although traditional dietary differences between Muslims and Hindus may be a factor, with the latter group shunning animal protein, lower income levels and health care use among Hindus in Bangladesh may be another explanation [53].

According to our analysis, women from higher socioeconomic groups are taller, reinforcing the positive association between income and height. Studies on adult height in India also showed that the average height of those belonging to Scheduled Tribe and Scheduled Caste categories were shorter than those of higher castes. Over time, the average height of Indians for both males and females has improved, but the improvement was higher among males than females [54].

The series of BDHS has consistently found regional variations in indicators of health and health care use in Bangladesh. The eastern divisions in the country have historically had higher fertility and lower contraceptive prevalence rates than the rest. The west of the country, bordering West Bengal, continues to exhibit lower fertility rates and higher contraceptive prevalence rates than the average for the country. This regional variation has been explained by the diffusion theory, where women in the West, many of whom may have migrated from India to Bangladesh since the Partition of 1947, behave more like women in West Bengal due to the physical proximity and, therefore, the diffusion of ideas and practices [55]. This may explain why women in the western divisions of Bangladesh were found to be relatively taller than the national average. On the other hand, although early marriage and childbearing in the Western region were likely to disfavour women from being taller than women in other regions, it was otherwise true [56]. Better childhood nutrition in the western region may have overpowered the adverse effect of early childbearing [56].

Last, our analysis found early childbearing associated with growth retardation among women. Studies show that shorter women give birth to babies with low birth weight [57]. There is also evidence that babies born to stunted women are at a greater risk of dying than children born to women of normal height [49]. The close relationship between mother’s and her child’s health/height underscores that stunting among children cannot be improved before addressing mother’s health first. A study in Nepal came to similar conclusions; both early marriage and early pregnancy were independently associated with shorter stature, accounting for a decrement of 1.4 cm, which decreased to 1 cm after adjusting for women's education [58].

### Strengths and limitations

To the best of our knowledge, this is the first study to examine the change in women’s height over time and the effect of teen childbearing in the Bangladeshi population. We used multiple nationally representative survey data and adopted appropriate statistical methods to ensure robust estimates. However, the study also has a few limitations, mainly due to the secondary data source. The BDHS collected anthropometric data from ever-married women ages 15-49, limiting the scope of including never-married women in the analysis. The analytic sample included women aged 20-29 years. Thus, we excluded nine percent of the never-married women in this age group; however, this likely did not affect our findings, because this was a small proportion, and the average height of the excluded women was also likely to increase over time.

The other important limitation is that we did not know the childhood nutritional status, residence, and wealth quintiles of women’s parental households (urban/rural residence and geographical region), which were factors of their height. We used the residence and wealth quintiles of the women’s in-laws’ households as proxies. The BY is the proxy of the women’s childhood nutrition; women born in a year had better nutrition than women born in the previous year. However, the proxy measures were the source of misclassification to some extent.

Another limitation is the various arguments about the minimum age of reaching full height. Deaton [47] discussed that South Asian women do not reach adult height until early 20s, but we included women age 20-29. While women in their early 20s may still grow a little, this most likely did not affect our results. Moreover, we performed a separate analysis including women age 25-29 on the survey day, and had similar findings to those for women age 20-29 on the survey day (results not shown).

Additionally, we excluded 1.4% of the women aged 20-29 years due to missing height data, and 0.15% of the height data to avoid extreme value noise. However, such a small portion of the data was unlikely to affect any estimates. Moreover, the background characteristics of the excluded and included women were similar (data not shown).

Finally, there were methodological issues related to sample size and power and mathematical tests of normality of height data—none of the BDHSs estimated sample sizes for analysing women’s height. Moreover, we examined women’s height by BY, not by BDHS round. So, we calculated the required minimum sample sizes by BY and found that the sample sizes were large enough to estimate the mean height for 21 out of 25 BY groups (**Table 2**). We did not perform any power analysis because post hoc power analysis has been studied to be conceptually flawed and analytically misleading [59]. Second, we did not use mathematical methods like the Shapiro-Wilk test, Shapiro-Francia test, or skewness/kurtosis for examining the normality of height data, as they were primarily developed for diagnosing small sample data normality and are not appropriate for large data.

## CONCLUSIONS

There has been little research done on female adult height. Evidence on adult height has largely come from developed societies using genetics and environmental factors, such as nutrition and income, as the primary drivers determining height differences across race and region. South Asian women continue to be among the shortest in the world. The height of the average Bangladeshi woman is 151 cm, one of the lowest in the world. There is need for in-depth research on the determinants of female adult height in South Asia, especially considering socio-cultural customs. Traditional and adverse practices, such as teenage pregnancy, may have prevented Bangladeshi women from being taller than they are today. About a third of Bangladeshi women continue to start childbearing around age 16 when they are still not fully physically developed themselves. Such practices perpetuate unfavourable health outcomes for mothers and their children. It is imperative to design and implement robust strategies to delay childbearing and focus on the health of mothers to ensure children's health. The Sustainable Development Goal targets for lowering child stunting by 2030 cannot be achieved without improving the health of mothers, first by delaying their first birth, among other factors. Further research is needed for an in-depth understanding of the reasons behind the slower improvement in height among sub-groups, such as non-Muslims and those living in the eastern part of the country, so that tailor-made interventions can be implemented.

## Additional material


Online Supplementary Document
