# Supplementary material for: Trends in women’s height and the effect of early childbearing on height retardation: An analysis of the height of Bangladeshi women born between 1974 and 1998
Source: J Glob Health. 2023 Sep 29;13:07006. doi: 10.7189/jogh.13.07006 (PMC10534193; doi:10.7189/jogh.13.07006)
Supplement: Online Supplementary Document [file jogh-13-07006-s001.pdf]

2023 ● Vol. 13 ● 07006 doi: 10.7189/jogh.13.07006

Trends in women's height and the effect of early childbearing on height retardation: An analysis of the height of Bangladeshi women born between 1974 and 1998

M Moinuddin Haider ,[1] Nahid Kamal, [2] Shusmita Khan ,[3] Mahabubur Rahman ,[1] Nayem Dewan ,[4] Sadman Sowmik Sarkar ,[1] Sabit Saad Shafiq ,[1] Nurul Alam ,[1]

[1]International Centre for Diarrhoeal Disease Research, Dhaka, Bangladesh
[2]PopDev Consultancy Ltd., London, UK
[3]Data for Impact, University of North Carolina at Chapel Hill, USA
[4]Bangladesh Bank, Dhaka, Bangladesh

Appendix A1. Household wealth quintiles of brides and grooms married in 2014, Matlab Health and Demographic Surveillance System.

The Matlab HDSS records marriage, divorce, other demographic events, and selected health information through two-monthly household visits. The HDSS also conducts periodic socio-economic censuses (SEC), and the HDSS collected data on the economic conditions of both bride and groom's households are available if both are from the HDSS area [1]. Of the 3,365 marriages in the HDSS area in 2014, when the HDSS conducted the last SEC, 474 brides and grooms were residents of the HDSS area. Among the remaining 2,891 marriages, either the bride or the groom was from outside the HDSS area.

Table A1. Number of brides and grooms by their HWQ groups, 2014 Matlab HDSS

	HWQ of the groom			
HWQ of the bride	Lowest	Middle three	Highest	Total
Lowest	**34**	19	1	54
Middle three	15	**266**	38	319
Highest	2	28	**71**	101
Total	51	313	110	474

We examined the 2014 household wealth quintiles (HWQs) of 474 brides and grooms who were residents of the HDSS area. Instead of the five categories, we used three: the lowest, the middle three, and the highest. **Table A1** shows that 78% [{(34 + 266 + 71) / 474} × 100] of the brides and grooms were from the same wealth groups.

Appendix 2. Statistical characteristics of the height of women born between 1974 and 1998

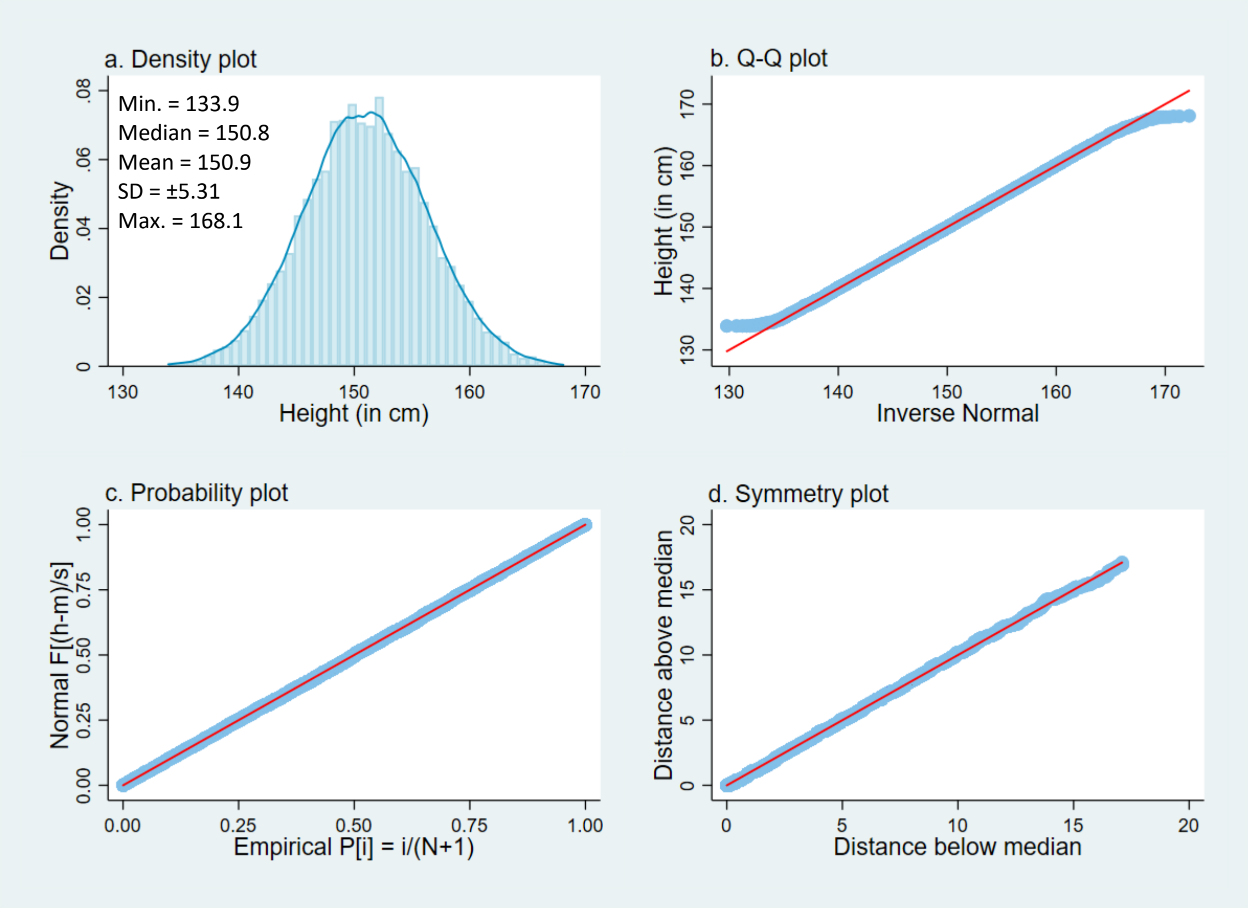

References

1. Alam N, Ali T, Razzaque A, Rahman M, Zahirul Haq M, Saha SK, Ahmed A, Sarder AM, Moinuddin Haider M, Yunus M, Nahar Q. Health and demographic surveillance system (HDSS) in Matlab, Bangladesh. International Journal of Epidemiology. 2017 Jun 1;46(3):809-16. https://doi.org/10.1093/ije/dyx076
